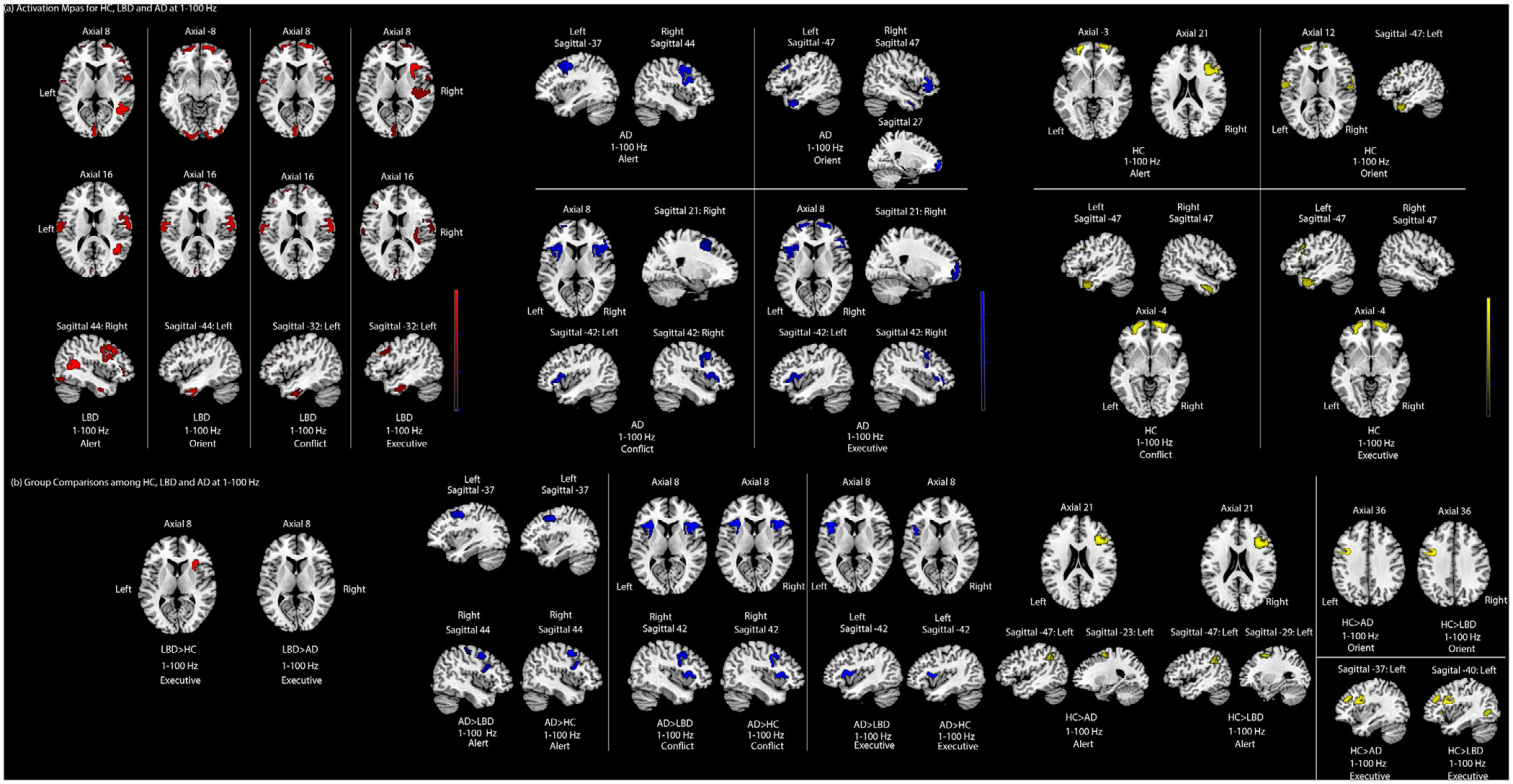# Attention Network Dysfunctions in Lewy Body Dementia and Alzheimer's Disease

**DOI:** 10.1002/alz.093622

**Published:** 2025-01-09

**Authors:** Li Su

**Affiliations:** ^1^ University of Sheffield, Sheffield United Kingdom

## Abstract

**Background:**

Attention deficits are notable in Lewy body dementia (LBD) and in Alzheimer’s disease (AD), however, its underlying neurobiology and neuropathology are unclear. Functional magnetic resonance imaging (fMRI) and electroencephalograph (EEG) provides information about attention deployment and regional neural oscillatory deficits in LBD and AD. In this study, we combined fMRI and EEG to detect neural correlates of attention dysfunctions in LBD and AD.

**Method:**

We recruited 33 patients with LBD, 15 patients with AD and 19 elderly healthy controls. All participants underwent a 3T fMRI scanning and a subsequently EEG recording. During fMRI and EEG, participants performed the same modifiedAttention Network Task (ANT) to investigate the alert, orient, executive and conflict attention dysfunctions. Here, we have focused on the EEG part of the study while using the fMRI data to localise the EEG signal on the cortex.

**Result:**

Behaviourally, although both LBD and AD maintained a relative normal executive/conflict attention, we found that LBD had alert attention deficits and AD showed apparent orient attention dysfunctions. Based on source‐level EEG analyses, LBD had frontal‐central deficits for alert attention while AD showed inferior frontal and precentral impairments for orient attention. In addition, the insular and inferior frontal areas was hyper‐activated in LBD and AD for executive/conflict attention. Apart from these areas, LBD showed complementary temporal‐central‐occipital network for the modified ANT task. Furthermore, the oscillational sources for the ANT effects indicated that alpha and theta bands were partly impaired in dementia patients.

**Conclusion:**

In summary, using source localised EEG, we found that attention dysfunctions in LBD and AD have engaged different neural networks. This has important implications in understanding the symptomology in LBD such as recurrent visual hallucinations and cognitive fluctuation.